# Characterization of genes related to the efflux pump and porin in multidrug-resistant *Escherichia coli* strains isolated from patients with COVID-19 after secondary infection

**DOI:** 10.1186/s12866-024-03283-8

**Published:** 2024-04-10

**Authors:** Aryan R. Ganjo, Salah Tofik Jalal Balaky, Ahang Hasan Mawlood, Sakar B. Smail, Nazar P. Shabila

**Affiliations:** 1 Department of Clinical Analysis, College of Pharmacy, Hawler Medical University, Erbil, Kurdistan Region Iraq; 2 Department of Medical Analysis, Faculty of Applied Science, Tishk International University, Erbil, Iraq; 3 Department of Medical Microbiology, College of Health Sciences, Hawler Medical University, Erbil, Kurdistan Region Iraq; 4 Department of Medical Laboratory Technique, College of Medical Technology, AL-Kitab University, Kirkuk, Iraq; 5 Par Hospital, Erbil, Kurdistan Region Iraq; 6 College of Health Sciences, Catholic University in Erbil, Erbil, Kurdistan Region Iraq; 7 Department of Community Medicine, College of Medicine, Hawler Medical University, Erbil, Iraq

**Keywords:** Antibiotic resistance, CCCP, COVID-19, *Escherichia coli*, Efflux pumps

## Abstract

**Background:**

*Escherichia coli* (*E. coli*) is a multidrug resistant opportunistic pathogen that can cause secondary bacterial infections in patients with COVID-19. This study aimed to determine the antimicrobial resistance profile of *E. coli* as a secondary bacterial infection in patients with COVID-19 and to assess the prevalence and characterization of genes related to efflux pumps and porin.

**Methods:**

A total of 50 nonduplicate *E. coli* isolates were collected as secondary bacterial infections in COVID-19 patients. The isolates were cultured from sputum samples. Confirmation and antibiotic susceptibility testing were conducted by Vitek 2. PCR was used to assess the prevalence of the efflux pump and porin-related genes in the isolates. The phenotypic and genotypic evolution of antibiotic resistance genes related to the efflux pump was evaluated.

**Results:**

The *E. coli* isolates demonstrated high resistance to ampicillin (100%), cefixime (62%), cefepime (62%), amoxicillin-clavulanic acid (60%), cefuroxime (60%), and ceftriaxone (58%). The susceptibility of *E. coli* to ertapenem was greatest (92%), followed by imipenem (88%), meropenem (86%), tigecycline (80%), and levofloxacin (76%). Regarding efflux pump gene combinations, there was a significant association between the *acrA* gene and increased resistance to levofloxacin, between the *acrB* gene and decreased resistance to meropenem and increased resistance to levofloxacin, and between the *ompF* and *ompC* genes and increased resistance to gentamicin.

**Conclusions:**

The antibiotics ertapenem, imipenem, meropenem, tigecycline, and levofloxacin were effective against *E. coli* in patients with COVID-19. Genes encoding efflux pumps and porins, such as *acrA, acrB,* and outer membrane porins*,* were highly distributed among all the isolates. Efflux pump inhibitors could be alternative antibiotics for restoring tetracycline activity in *E. coli* isolates.

## Background

Coronavirus disease 2019 (COVID-19) is an acute respiratory infection that can potentially cause serious complications caused by severe acute respiratory syndrome coronavirus 2, known as severe acute respiratory syndrome coronavirus 2 (SARS-CoV-2) [1]. The severity of the disease in SARS-CoV-2-infected patients spans a wide range and has spread throughout the world, making it a severe threat to public health from a carrier state without obvious symptoms to a life-threatening case of severe respiratory tract infection [2].

A secondary bacterial infection could result in a lower discharge rate and increased mortality [3]. Critically ill patients with COVID-19 are at high risk of secondary bacterial infection, such as secondary pneumonia, ventilator-assisted pneumonia, bloodstream infections, and even device-related secondary infections, during prolonged stays in intensive care units [4]. It has been hypothesized that patients with COVID-19 in critical care are at an even greater risk of developing secondary infections due to immunosuppression caused by the virus or drugs [5].

Determining the causal microorganisms responsible for secondary bacterial infections in critically ill patients with COVID-19 presents additional opportunities for investigation, such as empirical antibiotic treatment specifically designed for patients exhibiting initial signs of a secondary infection. Identifying potential risk factors related to secondary bacterial infections could help create novel techniques for prevention [6, 7].

The prevalence of secondary bacterial infection is approximately 10%-15% and is most common in coinfections and secondary infections [8]. *Escherichia coli (E. coli)* is one of the most common infectious disease agents and an important public health risk posed by alarmingly high rates of antibiotic resistance [9, 10]. The development of antibiotic resistance is a complicated and multifaceted process [11]. However, this process evolves due to prolonged exposure to antibiotics. Selective pressure has led to the evolution of various genetic mechanisms [12]. The resistance of *E. coli* to various antibiotics results from a complex interplay of factors, such as the modification of antibiotic targets, activation of efflux pump systems, membrane impermeability, and release of antibiotic-inactivated enzymes [13, 14]. Within these vital mechanisms, efflux pump systems play an important role in developing multidrug resistance by increasing antibiotic transport outside the cell through efflux pumps and porins [15, 16].

Efflux pump protein-based structures play a crucial role in bacterial physiology by mediating the uptake and excretion of essential nutrients, toxic substances, metabolic waste, and decreased antibiotic influx into the cell through changes in cell wall structure [17]. Generally, bacterial efflux pump systems can be divided into six different groups: the cassette superfamily of the adenosine triphosphate-binding cassette superfamily; multidrug and toxic compound extrusion; the major facilitator superfamily; small multidrug resistance; the resistance nodulation division family, which can extrude aminoglycosides, fluoroquinolones, and B-lactams; and the proteasomal antimicrobial compound efflux family [14].

The AcrAB-TolC efflux pump, a member of the resistance nodulation division family, is the main efflux pump in virulent strains of *E. coli* [18]. This pump comprises three important parts: the periplasmic membrane fusion protein AcrA, the inner membrane protein transporter AcrB, and the outer membrane channel TolC [19]. The AcrAD-TolC efflux pump has been reported to play an important role in resistance to different groups of antibiotics, such as aminoglycoside, fluoroquinolone, tetracycline, β-lactam, and carbapenem antibiotics [20–22]. However, outer membrane porins are closely associated with antibiotic resistance because they facilitate the passive diffusion of antibiotics through the outer membrane. Moreover, *OmpC* and *OmpF* are nonspecific porins involved in antibiotic transport and membrane integrity [23].

Although numerous studies have examined the epidemiological and clinical characteristics of coronavirus disease, data on secondary bacterial infections have been scarce [24, 25]. Therefore, this study aimed to determine the antimicrobial resistance profile of *E. coli* as a secondary bacterial infection in COVID-19 patients from Erbil city, Iraq, and the prevalence of genes related to pump and porin efflux among the *E. coli* isolates studied.

## Methods

### Bacterial isolation and study design

Fifty *E. coli* isolates were collected from patients with COVID-19 pneumonia who were hospitalized in the intensive care unit of one hospital in Erbil city, Iraq, between December 2020 and November 2021. All patients with COVID-19 and secondary *E. coli* infection in the studied hospital were included. The Research Ethics Committee of the College of Pharmacy of Hawler Medical University approved the study. Due to the retrospective nature of the study and the urgency of collecting data during the pandemic, initial verbal informed consent was waived. Finally, written consent was obtained from participants at a later stage. These isolates were identified by conventional and biochemical tests and confirmed by Vitek 2 (bioMerieux, USA) (GN-card) [6, 26].

### Antibacterial susceptibility test

The susceptibility of the *E. coli* isolates to antibiotics was assessed by the Vitek 2 system (bio-Mérieux, Inc., Durham, NC, USA) based on AST-GN69 and the AST-XN06 cards according to the manufacturer’s instructions. The antibiotics tested included ampicillin (AMP), amoxicillin-clavulanic acid (AMX-C), piperacillin/tazobactam, amikacin (AMK), gentamicin (GEN), meropenem (MEM), imipenem (IPM), ertapenem (ETP), ciprofloxacin (CIP), LEV, TG, trimethoprim-sulfamethoxazole, ceftazidime, cefepime (CFP), ceftriaxone (CFN), cefuroxime (CXM), and cefixime (CFX) [27].

### Efflux pump activity assay

The minimum inhibitory concentration (MIC) of tetracycline was calculated in 96-well plates using a broth microdilution method based on the Clinical and Laboratory Standards Institute Performance Standards for Antimicrobial Susceptibility Testing 2021 [28]. According to the manufacturer's instructions, the tetracycline powder was dissolved in sterilized deionized water. The concentrations used were then 256, 128, 64, 32, 16, 8, 4, 2, 0.5, and 0.25 µg/mL. Then, 100 µL of the produced dilutions were added to each well of the 96-well microplate. The microbial suspension was concentrated to 0.5 McFarland standard and diluted at a ratio of 1/100. The plates were then incubated at 35 °C for 18 - 24 h, with 100 µL of microbial suspension was added to each well. The MIC was defined as the lowest antibiotic concentration that prevented growth [29]. A final concentration of 12 µg/mL carbonyl cyanide 3-chlorophenylhydrazone (CCCP) inhibitor was added to each Muller Hinton broth containing 0.25 - 256 µg/mL tetracycline. This concentration was used as a sub-MIC of CCCP after optimization by using several concentrations of CCCP to inhibit bacterial isolates. Tetracycline was used as an indicator for genes related to efflux, as previously described [30]. After CCCP therapy, the antibiotic MICs were measured once more. The fourfold decrease in the MIC after the application of the CCCP inhibitor was interpreted as an indicator of substantial efflux pump activity. The control sample was a CCCP-containing, antibiotic-free plate [31].

### Molecular analysis

The strains were stored at -70 °C in 20% (vol/vol) glycerol in BHI medium and grown overnight on MacConkey agar at 37 °C to retain genetic variation. A bacterial colony was suspended in 500 µl of distilled water. The DNA extraction kit (G-nad and Beta Bayern) was used as directed by the manufacturer. The DNA was stored at -20 °C until needed. The primer sets to detect key efflux pump genes (Table 1) were used as previously described [32–34].

**Table 1:** Primer sequences, product sizes, and annealing temperatures of target genes of the isolates used in this study

**Genes**	**Primer sequences** (**5′-3′)**	**Product size (bp)**	**Annealing temperature (°C)**	**References**
*acrA*	F: ggtcgttctgatgctctca	1078 bp	52 °C	[35]
	R: ggcttgctggttattatcag			
*acrB*	F: cgtctaacagtgactccacgg	2730 bp	52 °C	[35]
	R: ttcaatcagacctttaccttc			
*tolC*	F: atgcaaatgaagaaa	100 bp	49 °C	[35]
	R: ttaatgacggaacggatt			
*ompF*	F: ggtctgcgtccgtccat	99 bp	60 °C	[36]
	R: ggttgcgcccacttca			
*ompC*	F: aagtagtaggtagcaccaacatca	163 bp	60 °C	[36]
	R: gggcgaacaaagcacagaa			

For PCR, 3 µl of entire genomic DNA (5 µl of nucleus-free water, 10 µl of master mix, and 1 µl per primer) was used as a template. A standard PCR amplification program was used on a thermocycler (Eppendorf, M Germany) as follows: initial heating to 95 °C for 5 min, followed by 30 amplification cycles. Each cycle consisted of three phases: denaturation at 95 °C for 30 s, annealing at 56 °C for 30 s, and extension at 72 °C for an adequate time, followed by a final 5-min step of elongation at 72 °C. Finally, the PCR products were stored at 4 °C until further analysis. The PCR products were electrophoresed on a 1% agarose gel, stained with ethidium bromide, and visualized using a UV transilluminator.

### Statistical analysis

Continuous data are shown as mean ± standard deviation (SD). Categorical data were analyzed using Chi-square and Fisher's exact tests and are presented as frequencies and percentages. A* P* value < 0.05 was considered to indicate statistical significance.

## Results

The preliminary identification of the 50 *E. coli* strains from the sputum sample of COVID-19 patients as secondary bacterial infections was carried out according to their morphological and biochemical characteristics. Then, all the isolates were confirmed using the Vitek 2 system. The mean ± SD age of the patients was 48.9 (18.1) years (range 14-90 years). Most of the patients were 41-50 years old (34%), male (58%), and from urban areas (78%), as shown in Table 2.

**Table 2:** Descriptive characteristics of the study sample

**Characteristic**	**No**	**%**
Age (mean ± SD)	48.9 (18.1)	
**Age group (years)**		
≤ 30	6	12.0
31-40	9	18.0
41-50	17	34.0
51-65	8	16.0
> 65	10	20.0
**Gender**		
Female	21	42
Male	29	58
**Area of residence**		
Urban	39	78
Rural	11	22

Table 3 shows the changes in the fold reductions of the positive isolates. A two-fold or greater reduction in the MIC of the antimicrobial agent when CCCP is added in place of the antibiotic is considered the criterion of significance. The greatest reduction in the MIC was 8 times and was observed for four isolates, followed by a 7 times reduction for only one isolate. The highest number of isolates (10) had a threefold reduction, followed by seven isolates with a fourfold reduction.

**Table 3:** Detection of efflux pump resistance to tetracycline in *E. coli*

**Stain no**	**MIC of tetracycline alone (µg/mL)**	**MIC of tetracycline in the presence of CCCP (µg/mL)**	**Fold reduction**
1	32	2	4
2	125	32	2
3	16	0.5	5
4	2	1	1
5	64	16	2
6	64	16	2
7	8	0.5	4
8	8	2	2
9	64	2	5
10	64	64	0
11	32	16	3
12	8	4	8
13	1	0.5	2
14	4	0.5	3
15	64	1	6
16	125	32	2
17	125	32	2
18	16	2	3
19	125	0.5	8
20	125	8	4
21	32	32	0
22	32	1	5
23	125	32	2
24	64	4	4
25	32	8	2
26	125	64	1
27	32	4	3
28	64	8	3
29	64	16	2
30	8	8	0
31	32	1	6
32	125	16	3
33	4	1	2
34	1	0.025	2
35	4	0.5	3
36	8	8	0
37	32	8	2
38	64	32	1
39	32	32	0
40	64	0.025	8
41	2	0.25	3
42	64	1	6
43	125	0.5	8
44	4	0.25	4
45	64	4	4
46	125	64	1
47	64	4	4
48	32	4	3
49	125	1	7
50	2	0.5	3

Table 4 shows the resistance rates the of *E. coli* isolates to a variety of antibiotics. *E. coli* isolates demonstrated high resistance to AMP (100%), CFX and CFP (62%), AMX-C and CXM (60%), and CFN (58%). The susceptibility of *E. coli* was highest for ETP (92%), followed by IPM (88%), MEM (86%), TG (80%), and LEV (76%).

**Table 4:** *E. coli* antibiotic resistance rate in patients with COVID-19

**Antibiotic**	**Susceptible**		**Intermediate**		**Resistance**	
	**No**	**%**	**No**	**%**	**No**	**%**
AMP	0	0	0	0	50	100
AMX-C	11	22.0	9	18.0	30	60.0
Piperacillin/tazobactam	31	62.0	8	16.0	11	22.0
AMK	31	62.0	3	6.0	16	32.0
GEN	32	64.0	3	6.0	15	30.0
MEM	43	86.0	5	10.0	2	4.0
IPM	44	88.0	4	8.0	2	4.0
ETP	46	92.0	1	2.0	3	6.0
CIP	35	70.0	9	18.0	6	12.0
LEV	38	76.0	4	8.0	8	16.0
TG	40	80.0	2	4.0	8	16.0
Trimethoprim/sulfamethoxazole	28	56.0	2	4.0	20	40.0
Ceftazidime	25	50.0	3	6.0	22	44.0
CFP	18	36.0	1	2.0	31	62.0
CFN	18	36.0	3	6.0	29	58.0
CXM	17	34.0	3	6.0	30	60.0
CFX	15	30.0	4	8.0	31	62.0

The data produced from this study showed that carbapenem antibiotics, both IMP and MEM, are still 2 of the best antibiotics against the pathogen under study, as they are highly effective antibiotics. Genomic data from this investigation showed that *acrA* (820 bp) was the most common gene detected in the isolates (74.0%), followed by *acr B* (890 bp) (64.0%), *ompC* (162 bp) (48%), *ompF* (191 bp) (32%), and *tolC* (655 bp) (24%), as shown in Table 5.

**Table 5:** Distribution of the efflux pump and porin genes among the *E. coli* isolates

**Gene expression**	**Yes**		**No**	
	**No**	**%**	**No**	**%**
*acr A*	37	74.0	13	26.0
*acr B*	32	64.0	18	36.0
*omp F*	16	32.0	34	68.0
*omp C*	24	48.0	26	52.0
*tol C*	12	24.0	38	76.0

*acrA* and *acrB* were the most common combinations of genes among the isolates (20%). The three (6%) isolates did not amplify any of the genes tested (Table 6).

**Table 6:** Distribution of efflux pump and porin gene combinations among *E. coli* strains

**Gene combination**	**No. of isolates**	**%**
*acr A* and *acr B*	10	20.0
*acr A, acr B, omp F, omp C*	6	12.0
*acr A*	6	12.0
*acr A, acr B, omp C*	5	10.0
*acr A, acr B, omp C, tol C*	3	6.0
*tol C*	2	4.0
*omp F, omp C, tol C*	2	4.0
*acr B, omp F, omp C*	2	4.0
*acr B*	2	4.0
*acr A, acr B, omp F, omp C, tol C*	2	4.0
*omp F, omp C*	1	2.0
*omp C*	1	2.0
*acr A, omp F, omp C, tol C*	1	2.0
*acr A, omp F, omp C*	1	2.0
*acr A, acr B, tol C*	1	2.0
*acr A, acr B, omp F*	1	2.0
*acr A, tol C*	1	2.0
*All negative*	3	6.0

Table 7 shows the associations between resistance to different antibiotics and the gene groups of *E. coli*. The *acrA* gene was significantly associated with increased resistance to levofloxacin (*P* = 0.022). The *acrB* gene was significantly associated with decreased resistance to meropenem (*P* = 0.006) and increased resistance to levofloxacin (*P* = 0.036). The *ompF* gene was significantly associated with increased resistance to gentamicin (*P* = 0.001). The *ompC* gene was significantly associated with increased resistance to gentamicin (*P* = 0.048). *ThetolC* gene was significantly associated with higher resistance to gentamicin (*P* = 0.004), meropenem (0.048), imipenem (*P* = 0.024), and tigecycline (*P* = 0.046).

**Table 7:** Association between resistance to different antibiotics and the gene groups

**Antibiotics**	**acr A (820 bp)**				**acr B (890 bp)**				**amp F (191 bp)**				**amp C (162 bp)**				**tol C (655 bp)**			
	**Positive**		**Negative**		**Positive**		**Negative**		**Positive**		**Negative**		**Positive**		**Negative**		**Positive**		**Negative**	
	**No**	**%**	**No**	**%**	**No**	**%**	**No**	**%**	**No**	**%**	**No**	**%**	**No**	**%**	**No**	**%**	**No**	**%**	**No**	**%**
**Amoxicillin-clavulanic acid**																				
Susceptible	9	24.3	2	15.4	7	21.9	4	22.2	4	25.0	7	20.6	5	20.8	6	23.1	3	25.0	8	21.1
Resistant	28	75.7	11	84.6	25	78.1	14	77.8	12	75.0	27	79.4	19	79.2	20	76.9	9	75.0	30	78.9
*P* value	0.704*				1.000*				0.728*				0.848				1.000*			
**Piperacillin/tazobactam**																				
Susceptible	23	62.2	8	61.5	22	68.8	9	50.0	9	56.3	22	64.7	14	58.3	17	65.4	7	58.3	24	63.2
Resistant	14	37.8	5	38.5	10	31.3	9	50.0	7	43.8	12	35.3	10	41.7	9	34.6	5	41.7	14	36.8
*P* value	1.000*				0.190				0.566				0.608				1.000*			
**Amikacin**																				
Susceptible	22	59.5	9	69.2	20	62.5	11	61.1	8	50.0	23	67.6	12	50.0	19	73.1	5	41.7	26	68.4
Resistant	15	40.5	4	30.8	12	37.5	7	38.9	8	50.0	11	32.4	12	50.0	7	26.9	7	58.3	12	31.6
*P* value	0.742*				0.923				0.230				0.093				0.171*			
**Gentamicin**																				
Susceptible	25	67.6	7	53.8	21	65.6	11	61.1	5	31.3	27	79.4	12	50.0	20	76.9	3	25.0	29	76.3
Resistant	12	32.4	6	46.2	11	34.4	7	38.9	11	68.8	7	20.6	12	50.0	6	23.1	9	75.0	9	23.7
*P* value	0.504*				0.750				0.001				0.048				0.004*			
**Meropenem**																				
Susceptible	33	89.2	10	76.9	31	96.9	12	66.7	13	81.3	30	88.2	21	87.5	22	84.6	8	66.7	35	92.1
Resistant	4	10.8	3	23.1	1	3.1	6	33.3	3	18.8	4	11.8	3	12.5	4	15.4	4	33.3	3	7.9
*P* value	0.357*				0.006*				0.666*				1.000*				0.048*			
**Imipenem**																				
Susceptible	34	91.9	10	76.9	30	93.8	14	77.8	13	81.3	31	91.2	21	87.5	23	88.5	8	66.7	36	94.7
Resistant	3	8.1	3	23.1	2	6.3	4	22.2	3	18.8	3	8.8	3	12.5	3	11.5	4	33.3	2	5.3
*P* value	0.173*				0.171*				0.370*				1.000*				0.024*			
**Ertapenem**																				
Susceptible	34	91.9	12	92.3	30	93.8	16	88.9	14	87.5	32	94.1	22	91.7	24	92.3	10	83.3	36	94.7
Resistant	3	8.1	1	7.7	2	6.3	2	11.1	2	12.5	2	5.9	2	8.3	2	7.7	2	16.7	2	5.3
*P* value	1.000				0.612*				0.584*				1.000*				0.240*			
**Ciprofloxacin**																				
Susceptible	26	70.3	9	69.2	22	68.8	13	72.2	10	62.5	25	73.5	18	75.0	17	65.4	9	75.0	26	68.4
Resistant	11	29.7	4	30.8	10	31.3	5	27.8	6	37.5	9	26.5	6	25.0	9	34.6	3	25.0	12	31.6
*P* value	1.000*				0.797				0.514				0.459				1.000*			
**Levofloxacin**																				
Susceptible	25	67.6	13	100.0	21	65.6	17	94.4	11	68.8	27	79.4	18	75.0	20	76.9	8	66.7	30	78.9
Resistant	12	32.4	0	0.0	11	34.4	1	5.6	5	31.3	7	20.6	6	25.0	6	23.1	4	33.3	8	21.1
*P* value	0.022*				0.036*				0.486*				0.874				0.448*			
**Tigecycline**																				
Susceptible	31	83.8	9	69.2	25	78.1	15	83.3	12	75.0	28	82.4	18	75.0	22	84.6	7	58.3	33	86.8
Resistant	6	16.2	4	30.8	7	21.9	3	16.7	4	25.0	6	17.6	6	25.0	4	15.4	5	41.7	5	13.2
*P* value	0.420*				0.730*				0.707*				0.490				0.046*			
**Trimethoprim/sulfamethoxazole**																				
Susceptible	18	48.6	10	76.9	16	50.0	12	66.7	7	43.8	21	61.8	12	50.0	16	61.5	6	50.0	22	57.9
Resistant	19	51.4	3	23.1	16	50.0	6	33.3	9	56.3	13	38.2	12	50.0	10	38.5	6	50.0	16	42.1
*P* value	0.077				0.254				0.231				0.412				0.631			
**Ceftazidime**																				
Susceptible	17	45.9	8	61.5	15	46.9	10	55.6	7	43.8	18	52.9	10	41.7	15	57.7	5	41.7	20	52.6
Resistant	20	54.1	5	38.5	17	53.1	8	44.4	9	56.3	16	47.1	14	58.3	11	42.3	7	58.3	18	47.4
*P* value	0.333				0.556				0.544				0.258				0.508			
**Cefepime**																				
Susceptible	12	32.4	6	46.2	11	34.4	7	38.9	6	37.5	12	35.3	9	37.5	9	34.6	4	33.3	14	36.8
Resistant	25	67.6	7	53.8	21	65.6	11	61.1	10	62.5	22	64.7	15	62.5	17	65.4	8	66.7	24	63.2
*P* value	0.504*				0.750				0.880				0.832				1.000*			
**Ceftriaxone**																				
Susceptible	12	32.4	6	46.2	11	34.4	7	38.9	5	31.3	13	38.2	8	33.3	10	38.5	4	33.3	14	36.8
Resistant	25	67.6	7	53.8	21	65.6	11	61.1	11	68.8	21	61.8	16	66.7	16	61.5	8	66.7	24	63.2
*P* value	0.504*				0.750				0.631				0.706				1.000*			
**Cefuroxime**																				
Susceptible	11	29.7	6	46.2	11	34.4	6	33.3	6	37.5	11	32.4	9	37.5	8	30.8	4	33.3	13	34.2
Resistant	26	70.3	7	53.8	21	65.6	12	66.7	10	62.5	23	67.6	15	62.5	18	69.2	8	66.7	25	65.8
*P* value	0.322*				0.941				0.720				0.616				1.000*			
**Cefixime**																				
Susceptible	9	24.3	6	46.2	8	25.0	7	38.9	6	37.5	9	26.5	9	37.5	6	23.1	4	33.3	11	28.9
Resistant	28	75.7	7	53.8	24	75.0	11	61.1	10	62.5	25	73.5	15	62.5	20	76.9	8	66.7	27	71.1
*P* value	0.170*				0.304				0.514*				0.266				1.000*			

^*^Fisher’s exact test

## Discussion

Previous viral outbreaks and pandemics were associated with increased morbidity and mortality primarily due to secondary bacterial infections. Most of the patient deaths during the 1918–1919 influenza pandemic were caused by secondary bacterial infections rather than viral infections [37]. With symptoms ranging from a mild flu-like illness to acute respiratory distress syndrome, COVID-19 generally manifests as a respiratory tract infection. The causes of bacterial and fungal coinfection in hospitalized patients with COVID-19 are unclear. Fifteen percent of hospitalized patients in Wuhan experienced secondary bacterial infections related to COVID-19; these infections were more common in non-survivors than in survivors [38]. More secondary bacterial and fungal infections, particularly infections caused by *Haemophilus influenzae*, *Streptococcus pneumoniae*, *Staphylococcus aureus*, and *Aspergillus spp*., have been reported during viral (influenza) pneumonitis epidemics. These infections are commonly associated with poor patient outcomes [39]. Gram-negative bacilli, primarily *Pseudomonas aeruginosa, Enterobacter* species, and *E. coli,* were shown to be the most prevalent bacteria that cause secondary pneumonia in patients with COVID-19 according to a major multicenter study conducted in 36 European intensive care units [40].

This study revealed that COVID-19 patients with microbiologically examined infections had *E. coli* bacteria, consistent with a study finding that 7.0% of COVID-19 patients had secondary bacterial infections caused by *E. coli* [41]. According to the current study, *E. coli* isolates are extremely susceptible to carbapenem antibiotics, including IPM, MEM, and ETP (88, 86, and 92%, respectively). However, they demonstrated complete resistance to AMP, 60% resistance to AMX-C acid, and significant resistance to cephalosporins, GEN, and AMK. According to other studies, ESBL-producing bacteria are known to produce a significant level of antibiotic resistance in *E. coli* [42, 43].

A key element contributing to the increase in antimicrobial resistance is the inappropriate prescription of antibiotics. Among several chemicals used as efflux pump inhibitors, phenylalanine arginine-naphthylamide or CCCP are the most commonly used to assess the effect of the AcrAB efflux pump on drug resistance in *E. coli* strains [44]. This study evaluated the MICs of tetracycline with and without CCCP as an AcrAB efflux pump inhibitor. Approximately 90% of the *E. coli* isolates were cultured in the presence of CCCP and became less resistant to tetracycline.

A considerable loss in antibacterial activity (four to eightfold reduction) was reported in 18 (36% of isolates), indicating that the efflux mechanism plays an important role in resistance to tetracyclines. In agreement with our results, Hao et al. reported that some *E. coli* strains were resistant to all prevalent antibiotics except TG, including B-lactams, tetracycline, fluoroquinolones, aminoglycosides, polymyxin B (8 g / mL) and fluoroquinolones [45]. They showed that after adding CCCP at 10 g / ml, the MIC of GEN was reduced by twofold, while the MIC of MEM, tetracycline, CIP, and chloramphenicol decreased by fourfold. Meena et al. evaluated the multiple antibiotic resistance index and the resistance mechanism of multidrug-resistant *E. coli* isolates from chicken feces [46]. They reported that the four antibiotics they used in their study, ceftazidime, tetracycline, chloramphenicol, and streptomycin, all reduced the growth of all isolates when taken in conjunction with CCCP. However, no absolute efflux, or 100% reduction, was observed. A reduction in the amount of porins may cause this multidrug phenotype, together with other mechanisms that restrict the entry of antibiotics into the bacterial cell. As a result, it was established that active efflux pumps play a role in the antibiotic resistance process. This finding is also consistent with the findings of a study conducted in Ohio, USA, which reported that after growing *E. coli* isolates for several generations in increasing concentrations of CCCP when evolved strains were grown in the presence of different antibiotics, one of the strains was very sensitive to tetracycline and chloramphenicol due to a mutation in the *acrB* gene detected by whole genome sequencing [47]. In contrast, other evolved strains showed mutations in the multidrug-resistant components *acrAB* and *ybhR*. Similarly, another study reported that antibiotic activity was induced in the presence of CCCP (10 mg/L final concentration). CCCP restored the activity of tetracycline against *Klebsiella* spp., and the MIC of the bacteria decreased from 512 mg/L to 53.15 mg/L [48]. CCCP has been reported to deter the proton motive force through its effect on the transmembrane potential and the transmembrane pH [49]. Thus, bacterial cells are metabolically deactivated. It is not clear whether the synergism of CCCP with a variety of antibiotics is caused by bacterial cell metabolic deactivation or efflux pump deactivation. CCCP could restore tetracycline activity against *Klebsiella* spp. and *Helicobacter pylori* [50].

It has been proposed that CCCP causes the formation of metabolically inert cells, resulting in synergistic effects with antibiotics. Due to this and cellular toxicity to mammalian cells, CCCP has been restricted to laboratory use only [51]. Another barrier that efflux pump inhibitors may face in becoming a viable treatment option is the unavailability of preclinical and clinical data. There is a paucity of information available on in vivo models and patient data to complement efflux pump inhibitor activity. Nevertheless, CCCP is the most widely used laboratory efflux pump inhibitor [50]. Multidrug efflux pumps are critical for developing resistance to many antibiotic classes [52]. They function at the frontline to protect bacteria against antibiotics by reducing the intracellular concentration of drugs [53].

This study showed that most of the genes related to the efflux pump are present in *E. coli* isolates. *AcrA, acrB, tolC, omp F,* and *ompC* were found in 74% (37/50), 64% (32/50), 24% (12/50), 32% (16/50), and 48% (24/50) of the clinical isolates, respectively. Similarly, Fayyazi et al. 2020 reported that 78.4%, 89.2%, and 82.9% of the isolates were positive for *acrA, acrB,* and *tolC*, respectively [54]. The current investigation revealed that *acrA* and *acrB* were found in 20% of the clinical isolates tested, while *acrA, acrB, ompF,* and *ompC* were found in 12% of the clinical isolates. In contrary, investigations in Egypt revealed that *acrA* and *acrB* were present in 74.28% of the clinical isolates of uropathogenic *E. coli* examined [35]. Additionally, unlike findings from Iran, the copresence of *acrA-B* was found in 95.9% of the *E. coli* isolates [55]. As a result, new strategies for modulating these resistance nodulation division efflux pumps, such as AcrAB-TolC, are required since they are crucial in gram-negative bacteria and for removing hazardous chemicals and medications. For example, resistance nodulation division transporters play an important role in mediating bacterial resistance to antibiotics and heavy metals. Furthermore, a mutation in specific amino acid residues in strategic domains of these efflux pumps may represent a mechanism to reduce or inhibit the affinity of the efflux pump for their usual substrates, improving multidrug resistance [56]. Sulavik et al. (2001) created *E. coli* strains with null mutations in efflux pump genes. These findings agreed with the hypothesis that *tolC* contributes to intrinsic resistance with and without AcrAB. They also discovered that the overlapping substrate susceptibility profiles of the *acrAB* and *tolC* mutant strains support their interaction as a tripartite pump system [57]. *E. coli* K-12 has also been reported to have at least 20 multidrug efflux pump genes that confer resistance once overexpressed [56].

Porin has been shown to play a different role in membrane integrity, antibiotic resistance, or both. Many antibiotics, for example, use *ompF* as the primary mechanism of outer membrane penetration. However, *ompA* was significantly linked to membrane integrity, increasing the susceptibility of the *ompA* mutant to numerous antibiotics. *OmpC* was found to be important for both antibiotic resistance and membrane integrity [58]. It has been reported that genetic mutations can change the expression of major porins, resulting in a reduction in porin expression or complete loss of these porins. Porin loss is the main source of resistance to various antibiotics, particularly b-lactams [59]. Porins mediate the inflow of nutrients and several antimicrobial drugs in gram-negative bacteria, and the sensitivity of bacteria to antibiotics is greatly influenced by the porins’ permeability characteristics [23]. A recent study by Ejaz revealed the effect of porin loss on the development of resistance and suggested the mechanisms for such resistance development. It revealed that *Klebsiella pneumoniae* strains with OmpK35 and OmpK36 porin loss show conglomerate resistance mechanisms with AmpC and carbapenemases, resulting in the emergence of extensive drug resistance. This makes these strains resistant to many antibiotics, including carbapenem [59]. Carbapenem resistance is reportedly related to porin loss independent of carbapenemase production [60, 61]. Such extensive resistance related to these suggested mechanisms may pose serious therapeutic problems, with limited therapeutic options remaining in the future [59, 60].

The present study has a number of limitations. The study was conducted in a single hospital during the COVID-19 epidemic due to the lack of access to other hospitals. The types of patients, secondary bacterial infections, and antibiotic resistance might have been different in other patients and hospitals, limiting the generalizability of our findings. We assessed the MIC of only tetracycline, although the resistance profile showed resistance to many antibiotics, and similar studies have assessed the effect of CCCP on the MICs of several other antibiotics. It could be more helpful to increase the number of antibiotics tested and evaluate the effect of CCCP on the MICs of more antibiotics based on resistance profiles. This study did not use any control strains due to their unavailability in the Kurdistan Region of Iraq when conducting this study. This is another limitation of this study, which could impact the study outcomes.

## Conclusions

Secondary bacterial infection among patients with COVID-19 is a serious problem. With increasing antimicrobial resistance among these secondary infectious pathogens, infection control and antimicrobial stewardship measures must be reinforced. It has been concluded from the present study that *E. coli* isolates exhibit a high prevalence of resistance to the antibiotics ampicillin, cefixime, cefepime, amoxicillin-clavulanic acid, cefuroxime, and ceftriaxone. Our results indicate that efflux pumps and porins play a significant role in *E. coli* antibiotic resistance, which is worrisome and highlights the urgent need for future studies using efflux pump inhibitors in combination with antibiotics. Understanding the epidemiological patterns of secondary bacterial infection in patients with COVID-19 is critical for providing appropriate therapy and preventing the overuse of antibiotics for treating patients. The current study revealed that the antibiotics ertapenem, imipenem, meropenem, tigecycline, and levofloxacin were effective against *E. coli* in patients with COVID-19. Genes encoding efflux pumps and porins, such as *acrA*, *acrB*, and *omp,* were highly distributed among all the isolates. Efflux pump inhibitors could be alternative antibiotics for restoring tetracycline activity in *E. coli* isolates. More work is needed to identify new efflux pump inhibitors that could be used to treat such infections.

## Data Availability

All the data, including the values and numbers used to produce the results, are available upon reasonable request.

## Abbreviations

AMP: Ampicillin
AMX-C: Amoxicillin-clavulanic acid
CCCP: Carbonyl cyanide 3-chlorophenylhydrazone
CFN: Ceftriaxone
CFP: Cefepime
CFX: Cefixime
COVID-19: Coronavirus disease 2019
CXM: Cefuroxime
*E*. *coli*: *Escherichia coli*
ETP: Ertapenem
GEN: Gentamicin
IPM: Imipenem
LEV: Levofloxacin
MEM: Meropenem
MIC: Minimum inhibitory concentration
Omp: Outer membrane protein
PCR: Polymerase chain reaction
TG: Tigecycline
